# Synthesis of a Low-Molecular-Weight Filtrate Reducer and Its Mechanism for Improving High Temperature Resistance of Water-Based Drilling Fluid Gel System

**DOI:** 10.3390/gels8100619

**Published:** 2022-09-28

**Authors:** Xiaodong Dong, Jinsheng Sun, Xianbin Huang, Jian Li, Kaihe Lv, Pengxin Zhang

**Affiliations:** 1 School of Petroleum Engineering, China University of Petroleum (East China), Qingdao 266580, China; 2 Key Laboratory of Unconventional Oil & Gas, Development Ministry of Education, Qingdao 266580, China

**Keywords:** low-molecular-weight polymer, filtrate reducer, water-based drilling fluid gel system, high temperature resistance

## Abstract

During the exploitation of deep and ultradeep oil and gas resources, the high-temperature problem of deep reservoirs has become a major challenge for water-based drilling fluids. In this study, a novel high-temperature-resistant filtrate reducer (LDMS) with low molecular weight was synthesized using N, N-dimethylacrylamide; sodium p-styrene sulfonate; and maleic anhydride, which can maintain the performance of a drilling fluid gel system under high temperature. Unlike the conventional high-temperature-resistant polymer filtrate reducer, LDMS does not significantly increase the viscosity and yield point of the drilling fluid gel systems. After aging at 210 °C, the filtrate volume of a drilling fluid with 2 wt% LDMS was only 8.0 mL. The mechanism of LDMS was studied by particle size distribution of a drilling fluid gel system, Zeta potential change, adsorption experiment, change of bentonite interlayer spacing, filter cake scanning electron microscope, and related theoretical analysis. The mechanism study revealed that LDMS could be adsorbed on the surface of bentonite particles in large quantities and intercalated into the interlayer of bentonite. Thus, it can improve the hydration degree of bentonite particles and the colloidal stability of the drilling fluid gel system, maintain the content of fine particles in the drilling fluid gel system, form a compact mud cake, and significantly reduce the filtrate volume of the drilling fluid gel system. Therefore, this work will promote the application of a low-molecular-weight polymer filtrate reducer in high-temperature-resistant water-based drilling fluid gel systems.

## 1. Introduction

Oil is an indispensable fossil energy source for human production and life. After a long period of continuous exploitation, shallow as well as conventional oil and gas resources have been gradually depleted. Shallow oil and gas resources have been unable to meet the energy demand of the rapidly developing society. Therefore, the current focus of oil and gas development has been extended from shallow reservoirs to deep and ultra-deep reservoirs. Taking China as an example, the large oil and gas reservoirs discovered in recent years are mainly distributed in deep and ultra-deep formations, where the depth of the formation generally exceeds 6000 m and the temperature at the bottom of the well is close to 200 °C [1,2,3]. With the continuous increase of drilling depth, the situation of drilling into complex formations with high temperature and high pressure is also increasing. However, drilling fluids are extremely prone to lose efficacy in complex high temperature and high pressure environments, such as a dramatic increase in viscosity leading to the loss of fluidity, a decrease in yield point leading to a decrease in the rock-carrying ability, and a sharp increase in filtration loss. Severe deterioration of drilling fluid performance can also lead to major safety accidents such as well collapse, stuck drilling, well leakage and even well blowout [4]. Therefore, there is an urgent need to develop high temperature resistant drilling fluids suitable for deep and ultra-deep well drilling.

Drilling fluid is a circulating fluid used in the oil and gas drilling process and meet the needs of drilling work with its multiple functions. A drilling fluid plays an important role [5,6] in carrying and suspending rock cuttings, cooling and lubricating drilling tools, transmitting rock breaking power, flushing the bottom of the well, assisting in the collection and interpretation of formation information, stabilizing the wellbore and balancing formation pressure during the drilling process. Generally speaking, there are three types of drilling fluids that are more commonly used, water-based drilling fluids (WBDFs), oil-based drilling fluids (OBDFs) and synthetic-based drilling fluids (SBDFs). OBDFs [7,8] have good temperature resistance and lubricity with little damage to the oil and gas reservoirs. However, the base oil of OBDFs will produce toxic aromatic hydrocarbons during use, making the environmental acceptance of OBDFs poor. In order to make the drilling fluids have the excellent properties of OBDFs and good environmental acceptability, SBDFs [9,10] have come into being as an emerging family of drilling fluids. The continuous phase of SBDFs is synthetic organic matter, which leads to its high cost and limits its widespread application. WBDFs are most widely used because of their low price and environmental acceptability. Therefore, high-temperature-resistant WBDFs have always been the focus of research on a high-temperature-resistant drilling fluid. Water-based drilling fluid is a multiphase dispersion system, mainly composed of water, bentonite, weighting materials, and various functional additives (e.g., viscosifier, viscosity reducers, inhibitors, plugging agents, and filtrate reducers). During the drilling process, the drilling fluid will inevitably penetrate into the formation. The invasion of a drilling fluid filtrate into the formation can easily cause the hydration and expansion of the wellbore rock and destroy the stability of the wellbore. The filtration performance of a drilling fluid has an important influence on the protection of oil and gas formations and the stability of the wellbore. As the core additive of WBDFs, a filtrate reducer plays an important role in reducing the filtrate volume of a drilling fluid, maintaining the stability of drilling fluid performance, and ensuring drilling safety. Therefore, a filtrate reducer with excellent temperature resistance is an important factor to control the filtration property and maintain the stable performance of high-temperature-resistant drilling fluids.

Researchers have conducted massive research on improving the temperature resistance of filtrate reducers. At present, the filtrate reducers commonly used in the petroleum industry are mainly modified natural materials and synthetic polymers. Natural materials [11,12,13,14] (e.g., starch, cellulose, lignin, and xanthan gum) were the first to receive the attention of researchers due to their wide sources, low price, and environmental friendliness. However, due to the inherent characteristics of natural materials, their applicable temperature is usually below 150 °C, which cannot meet the needs of deep well drilling. Therefore, researchers have modified the natural materials by sulfonation, etherification, and grafting to improve their temperature resistance. Chang et al. [15] prepared an environmentally friendly anti-high-temperature filtrate reducer by grafting nanocellulose particles with amphoteric polymers through an intramolecular self-assembly method. The synthetic filtrate reducer could significantly improve the viscosity of a drilling fluid and reduce the filtrate volume. However, due to the poor temperature resistance of cellulose, the temperature resistance of the synthetic filtrate reducer did not improve significantly (still lower than 180 °C). In contrast, a synthetic polymer filtrate reducer can withstand much higher temperatures. Wang et al. [16] used N, N-dimethylacrylamide (DMAA), 2-acrylamide-2-methylpropanesulfonic acid (AMPS), N-vinylpyrrolidone (NVP), and sodium p-styrene sulfonate (SSS) as monomers to synthesize a high-temperature-resistant polymer filtrate reducer, whose temperature resistance was over 200 °C. The temperature resistance of the filtrate reducer was improved by introducing rigid groups in the side chain of the molecule to improve the rigidity of the molecular chain. Using AMPS, acrylamide (AM), SSS, and NVP, Li et al. [17] synthesized a polymer filtrate reducer with a temperature resistance of 200 °C. The special structure of NVP protected the adsorption groups (amide groups, cationic groups, etc.) from decomposition at high temperature, which enabled the filtrate reducer to adsorb on the bentonite surface, thereby improving its temperature resistance. Zhang et al. [18] synthesized a filtrate reducer with hydrophobic association properties using acrylamide, SSS, maleic anhydride (MAH), and lauryl methacrylate as monomers, with resistance to temperatures up to 200 °C. The filtrate reducer formed a network structure in the drilling fluid through the hydrophobic association of molecules, which significantly increased the viscosity and reduced the fluid loss of the drilling fluid at a high temperature. In addition, the application of nanomaterials in the synthesis of the filtrate reducer significantly improves the performance of drilling fluids. Nima Mohamadian et al. [19] synthesized a novel hybrid polymer nanocomposite poly (styrene-methyl methacrylate-acrylic acid)/nanoclay for drilling a fluid additive by microemulsion polymerization. Drilling fluids containing this terpolymer/nanoclay composite filtrate reducer had good rheology and fluid loss properties at 200 ℉ and reduced fluid loss by 50% for the bentonite-based fluids.

Synthetic polymeric filtrate reducers are all water-soluble polymers. After the high-molecular-weight polymeric filtrate reducers were dissolved in the drilling fluid, the molecular structure was fully stretched. On the one hand, the filtrate reducers were adsorbed on the surface of bentonite particles, and retained in the pores of the filter cake by bridging, which reduced the permeability of the filter cake and reduced the filter loss of the drilling fluid. On the other hand, the filtrate reducers reduced the filter loss by significantly increasing the viscosity of the drilling fluid through the formation of a gel network structure in the drilling fluid with their irregular molecular structure. The main mechanism [20,21,22] of the high-molecular-weight polymer filtrate reducer was to reduce the filtration by increasing the viscosity of the drilling fluid. Therefore, some scholars [23,24,25,26] have proposed that the larger the molecular weight of the water-soluble polymer filtrate reducer, the better the viscosity of the drilling fluid after high temperature aging. The higher the viscosity of the drilling fluid and the lower the fluid loss, the better the temperature resistance of the filtrate reducer. However, the drilling fluids need to use weighting materials to increase the density to balance the formation pressure. The deeper the formation, the higher the temperature and pressure at the bottom of the well, and the higher the density of the drilling fluid applied. With the increase in drilling fluid density, the viscosity and yield point also increase sharply. Adding high-molecular-weight polymer filtrate reducers to a high-density drilling fluid to control fluid loss will seriously affect the rheology of the drilling fluid. Additionally, drilling fluids with poor rheology are unusable. Here, a novel low-molecular-weight filtrate reducer (LDMS) was synthesized using DMAA, SSS, and MAH as monomers. LDMS does not significantly increase the viscosity and yield point of the drilling fluid, and has excellent fluid loss reduction performance. Our work can provide new insights and ideas for the study of a high-temperature-resistant polymeric filtrate reducer.

## 2. Results and Discussion

### 2.1. Characterization of LDMS

#### 2.1.1. Chemical Structure Analysis

**Figure 1:**
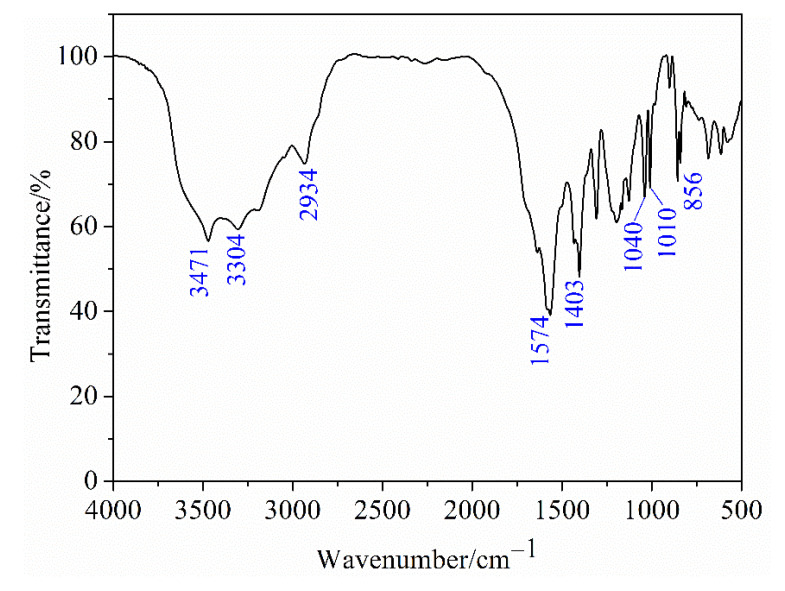
Infrared spectrum of LDMS.

The FTIR test results of LDMS are shown in Figure 1. The peaks at 3471 and 3304 cm^−1^ are –OH and crystal water in the product. The wavelength of 2934 cm^−1^ is the antisymmetric stretching vibration absorption peak of the C–H bond in the methyl group. The peaks at 1574 and 1406 cm^−1^ are the antisymmetric and symmetric stretching vibration absorption peaks of –COO^−^ in the carboxyl group. The peaks at 1040 and 1010 cm^−1^ are the symmetric stretching vibration absorption peaks of –SO_3_^−^ linked to the benzene ring. The out-of-plane bending vibration absorption peak of –CH on the benzene ring [16] affected by the para substituent in SSS appears at 856 cm^−1^. The FTIR characterization results showed that LDMS carried the characteristic functional groups of all the reacting monomers, and all the reacting monomers participated in the reaction successfully.

**Figure 2:**
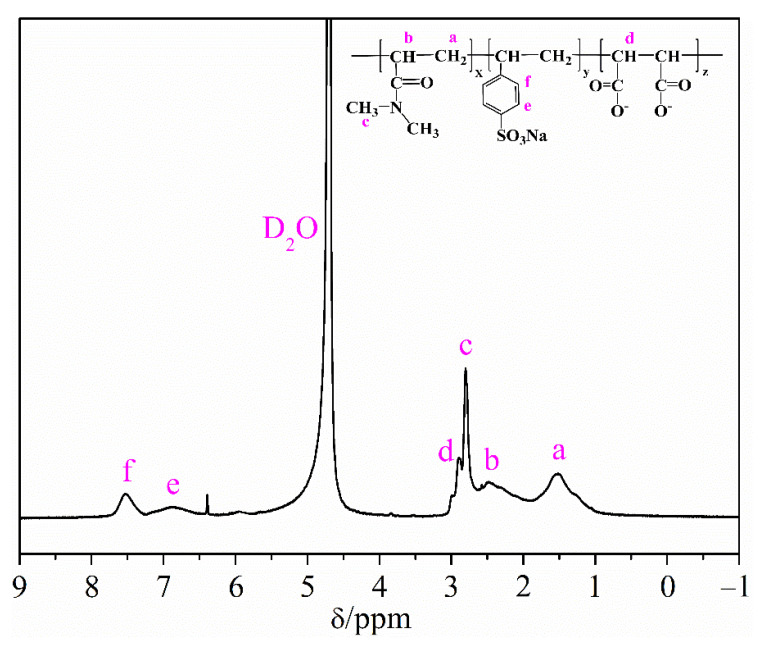
^1^H NMR curves of LDMS, (a–f represent hydrogen elements at different positions in the molecular chain).

The ^1^H-NMR of LDMS dissolved in heavy water is shown in Figure 2. Peak a (1.52 ppm) is the proton peak of –CH_2_ in the polymer backbone. Peak b (2.48 ppm) should be attributed to–CH– attached to the amide group on the main chain. The proton peaks of the two methyl groups in DMAA appear at peak c (2.80 ppm). Peak d (2.89 ppm) corresponds to the proton peak of –CH– attached to the carboxyl group on the main chain. The two unsubstituted hydrogen proton peaks on the benzene ring [18] in SSS can be observed at 6.86 ppm (peak e) and 7.52 ppm (peak f). The peak (4.70 ppm) is the solvent peak of heavy water D_2_O. The results of ^1^H-NMR also showed that the molecular structure of LDMS contained all the reacting monomers, and all the reacting monomers participated in the reaction successfully.

#### 2.1.2. Thermogravimetric Analysis

**Figure 3:**
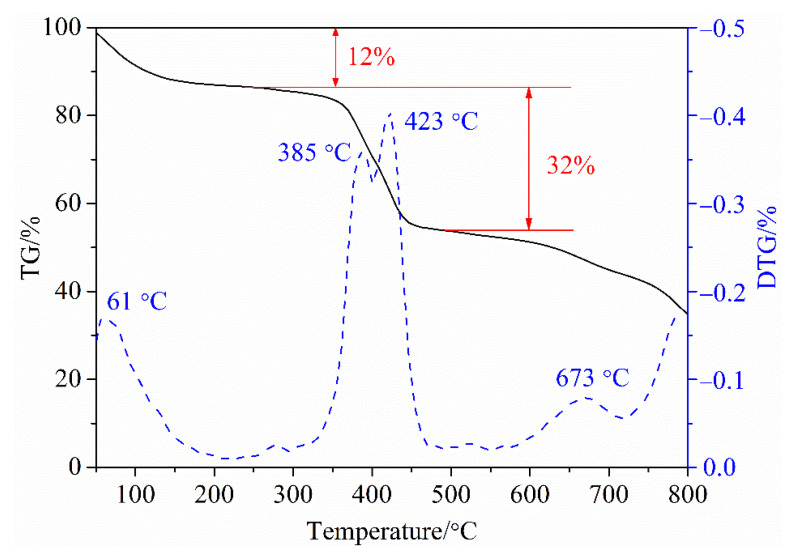
Thermogravimetric analysis curve of LDMS.

Drilling fluid additives are critical to maintaining the performance of drilling fluids under high temperature conditions, so a good thermal stability of additives is required. The thermogravimetric analysis curve (TG) and the derivative thermogravimetry curve (DTG) of LDMS are shown in Figure 3. The thermal decomposition process of LDMS can be divided into three stages. In the first stage, from 50 to 238 °C, the TG curve decreases gently with a weight loss of 12%, and the peak occurs at 61 °C. The mass loss in this stage is owing to the evaporation of free water in the sample and bound water adsorbed on the polymer. In the second stage, when the temperature ranges from 238 to 491 °C, there is a 32% thermal weightlessness platform, and the mass loss is noticeable. Two distinct peaks appear on the DTG curve at this stage. The peak at 385 °C is mainly related to the decomposition of the amide group [27] in the polymer side chain. The thermal decomposition rate reaches its highest at 423 °C due to the thermal decomposition of the sulfonic acid groups in the polymer and the C–C bonds of the polymer backbone. The third stage is after 491 °C, where the remaining part of the polymer continues to carbonize and decompose as the temperature increases. Thermal stability experiment shows that the polymer LDMS has good thermal stability, and the initial decomposition temperature is up to 238 °C. Generally, the depth of large oil and gas reservoirs buried in deep and ultradeep formations can reach 6000 m. The geothermal gradient is typically 30 °C/km, and at this depth, the bottom hole temperature is close to 200 °C. The thermal decomposition temperature of LDMS is higher than the bottom hole temperature, indicating that LDMS has good potential for application in high-temperature-resistant drilling fluids.

#### 2.1.3. Molecular Weight Determination

**Figure 4:**
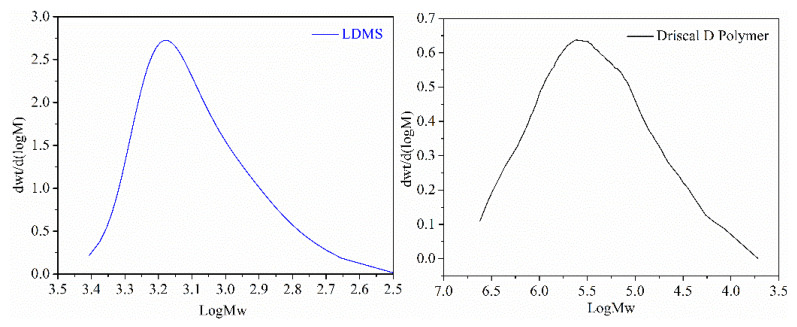
The molecular weight distribution of LDMS and Driscal.

In order to compare and investigate the effects of different molecular weight filtrate reducers in WBDFs, the relative molecular weights of LDMS and the purchased filtrate reducer Driscal D Polymer were first determined by GPC. The distribution of the relative molecular weights and the measurement results are shown in Figure 4 and Table 1. From the test results, it can be seen that the molecular weight distribution range of LDMS is narrow, and the polydispersity coefficient is 1.157, which is close to 1, indicating that its molecular weight distribution is concentrated. The purchased high-performance high-temperature-resistant filtrate reducer Driscal D Polymer has a large polydispersity coefficient of 5.797 because it is an industrial product. The weight-average molecular weight (M_w_) of LDMS is 1325, and that of Driscal D Polymer is 626,763. The M_w_ and number-average molecular weight (M_n_) of Driscal D Polymer are two orders of magnitude higher than those of LDMS. The Z-average molecular weight (M_z_) of Driscal D Polymer is three orders of magnitude higher than that of LDMS. In the chemical category, low-molecular-weight polymers [28] are polymers with small relative molecular weights ranging from 1 × 10^3^ to 6 × 10^3^. LDMS satisfies the relative molecular weight requirements of low-molecular-weight polymers, indicating that the low-molecular-weight filtrate reducer LDMS was synthesized successfully. At the same time, Driscal D Polymer was selected as the representative of high-molecular-weight polymeric filtrate reducers to conduct comparative experiments in WBDFs.

### 2.2. Performance of Filtrate Reducer in WBDFs

#### 2.2.1. Rheology and Filtration Performance Measurements

**Figure 5:**
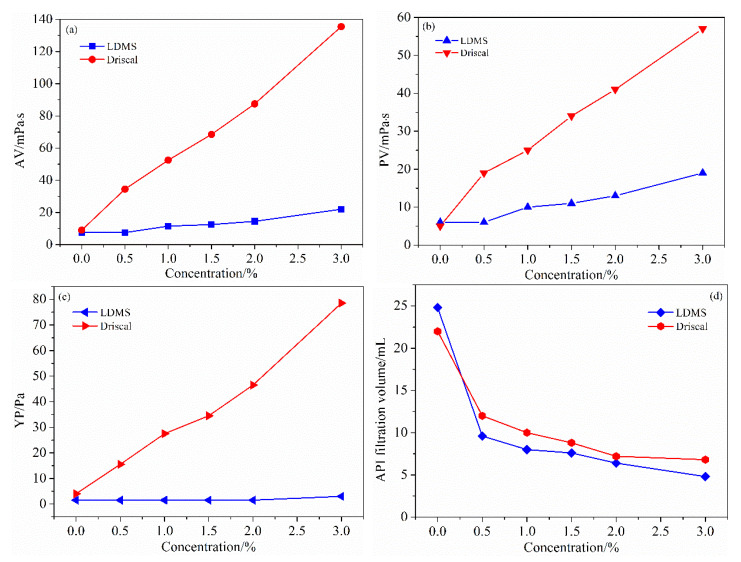
Effect of concentration on the AV (**a**), PV (**b**), YP (**c**), and API filtration (**d**) of drilling fluids.

In order to study the performance of LDMS in freshwater drilling fluids, different contents of LDMS and Driscal were added to the freshwater base fluid to prepare drilling fluids. The rheological parameters and API filtration of drilling fluids were measured at room temperature. The results of rheological parameters and filtration loss measurements are shown in Figure 5. With the increase in LDMS concentration, the AV (Figure 5a), PV (Figure 5b), and YP (Figure 5c) of the drilling fluid increased, and the API filtration (Figure 5d) decreased significantly. The viscosity of the drilling fluid added with 3 wt% LDMS was about three times that of the base fluid, the YP was about twice that of the base fluid, and the API filtration was reduced to one-fifth of that of the base fluid. With the increase in Driscal addition, the viscosity and yield point of the drilling fluid increased sharply, and the API filtration decreased. When 3 wt% Driscal was added to the base fluid, the AV was about 15 times that of the base fluid, the PV was about 11 times that of the base fluid, the YP was about 20 times that of the base fluid, and the API filtration was reduced to one-third of that of the base fluid. The increasing trend of the viscosity of the drilling fluid added with Driscal was much higher than that of the drilling fluid added with LDMS. Under the same concentration conditions, the viscosity of the Driscal-added drilling fluid was much higher than that of the LDMS-added drilling fluids, but the API filtration was higher than that of the drilling fluid with LDMS. It showed that LDMS does not only rely on the viscosity-increasing mechanism [20] of the filtrate reducer to reduce the filtrate volume of the drilling fluid. When the addition amount of LDMS was 3.0 wt%, the API filtration of the drilling fluid was only 4.8 mL, and the AV was only 22 mPa·s, indicating that LDMS has a good filtration loss reduction effect and does not significantly affect the rheological properties of drilling fluids.

#### 2.2.2. Temperature Resistance

**Figure 6:**
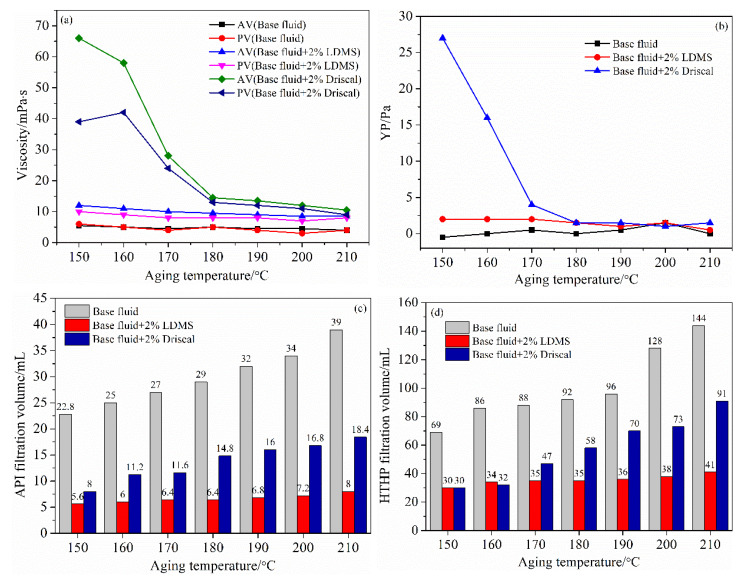
Effect of temperature on the viscosity (**a**), YP (**b**), API filtration (**c**), and HTHP filtration (**d**) of drilling fluids.

The drilling fluids were prepared by adding 2.0 wt% LDMS and 2.0 wt% Driscal to the freshwater base fluid. After the drilling fluids were aged for 16 h at different temperatures, the rheological parameters and API filtration were measured at room temperature. The HTHP filtration of the drilling fluid was measured at the corresponding aging temperature. The measurement results of rheological parameters and filtration are shown in Figure 6. The temperature resistance of LDMS was evaluated by the experimental results.

With the increase in the aging temperature, the AV, PV, and YP of the base fluid were very low and almost unchanged, and the API filtration and HTHP filtration increased sharply. The HTHP filtration of the base fluid at 210 °C was as high as 144 mL. It indicated that the gel network structure inside the base fluid had been destroyed. The viscosity and YP of the drilling fluid with LDMS decreased slightly with the increase in aging temperature. Compared with the base fluid, the viscosity and YP of the drilling fluid with LDMS were about twice that of the base fluid, which increased to a lesser extent, but the amount of filtration was significantly reduced. After aging at 210 °C, the API filtration of the drilling fluid with LDMS was only 8.0 mL, and the HTHP filtration was 41.0 mL. The viscosity and YP of the drilling fluid with Driscal changed dramatically with the increase in aging temperature. The viscosity and YP of the drilling fluid with Driscal decreased sharply with the increase in temperature before 180 °C, and the change was no longer significant after 180 °C, indicating that the temperature resistance turning point of Driscal is 180 °C. Compared with the base fluid, the viscosity and YP of the drilling fluid with Driscal increased greatly, and the filtrate volume also decreased. However, compared with the drilling fluid with LDMS, the drilling fluid with Driscal had a higher viscosity and yield point, and about twice as much the amount of filtration. After aging at 210 °C, the API filtration of the drilling fluid with Driscal was 18.4 mL, and the HTHP filtration was 91.0 mL.

The comparison results showed that the performance of Driscal was greatly affected by temperature. With the increase in temperature, the macromolecular chain of Driscal was gradually broken and decomposed, resulting in a decrease in performance. Additionally, the performance decreased significantly after 180 °C, indicating that the temperature resistance turning point for Driscal is 180 °C in a WBDF environment. However, with the increase in temperature, the performance of LDMS was very stable, indicating that the temperature resistance of LDMS is better than that of Driscal. This is because the small molecular chain of LDMS contains a rigid benzene ring, and the main chain is a carbon–carbon bond with large bond energy, which is not easy to break at high temperature, so that LDMS is not easily degraded at high temperature. In addition, LDMS can be firmly adsorbed on the surface of bentonite particles under the action of hydrogen bonding [29]. The thermal decomposition temperature of LDMS is as high as 238 °C, which is higher than the bottomhole temperature during the drilling process. Meanwhile, LDMS is not easily degraded at high temperature and desorbed from bentonite particles, which ensures the stability of the drilling fluid during drilling.

### 2.3. Mechanism Analysis

#### 2.3.1. Zeta Potential and Particle Size Analysis

**Figure 7:**
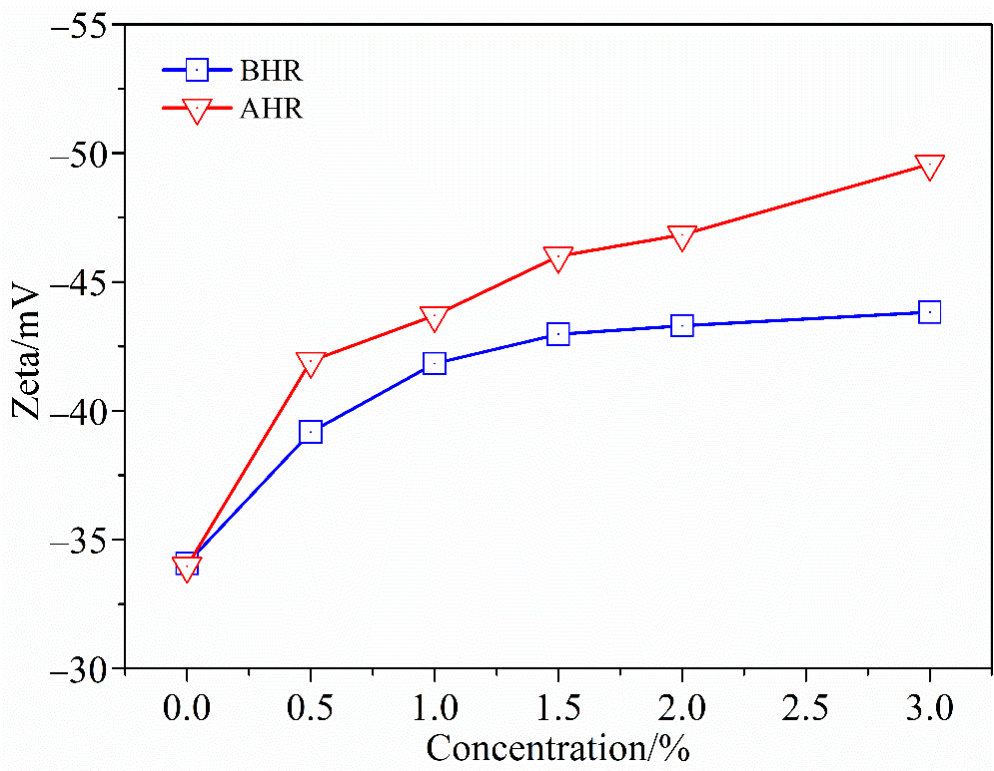
Changes of the Zeta potential of the drilling fluid before and after aging under different concentrations.

The drilling fluids were prepared by adding different contents of LDMS into the freshwater base fluid. The drilling fluids were aged at 180 °C for 16 h. The Zeta potential of the drilling fluid was measured before and after aging, and the results are shown in Figure 7. With the increase in the amount of LDMS, the absolute value of the Zeta potential of drilling fluids increased both before and after aging. After high-temperature aging, the Zeta potential of the drilling fluid without LDMS (base fluid) was changed from −34.07 to −33.97 mV, and the absolute value decreased. However, for the drilling fluid with LDMS, the absolute value of the Zeta potential after aging (AHR) was larger than that before aging (BHR). The Zeta potential of the drilling fluid is usually negative because of the negative charge on the surface of bentonite particles. The Zeta potential can reflect the stability of a drilling fluid dispersion system. The more stable the drilling fluid dispersion system is, the higher the degree of hydration of bentonite particles and the larger the absolute value of the Zeta potential. It is generally considered that the drilling fluid dispersion system is stable when the absolute value of the Zeta potential of the drilling fluid is larger than 30 mV [18,30]. The absolute values of the Zeta potential of drilling fluids with LDMS were all larger than 35 mV, indicating that the drilling fluid system has good stability and LDMS has a good gel protective effect. With the increase in LDMS addition, more and more small molecules of LDMS were adsorbed on the surface of bentonite particles to promote their hydration and made the hydration layer of bentonite particles thicker. The thickening of the hydration film of bentonite particles led to an increase in the negative charge on the bentonite particles and the absolute value of the drilling fluid Zeta potential. Normally, the hydration of bentonite particles is weakened after aging due to high temperature dehydration [31,32], and the hydration layer of bentonite particles becomes thinner, which leads to a decrease in the absolute value of the Zeta potential. However, in the drilling fluids containing LDMS, the situation is exactly the opposite, indicating that the presence of LDMS weakens the high temperature dehydration of the bentonite. This is a benefit from the good temperature resistance and gel protection ability of LDMS, which can still adsorb on the surface of bentonite particles even under high temperature conditions, maintain the hydration of bentonite particles, and maintain the stability of the drilling fluid colloidal dispersion system.

**Figure 8:**
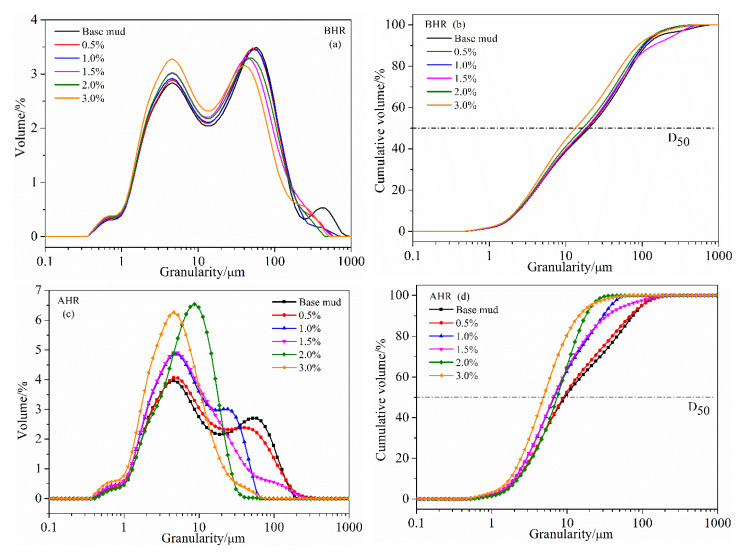
Particle size distribution of drilling fluids before and after aging: (**a**) granularity distribution BHR, (**b**) volume cumulative distribution BHR, (**c**) granularity distribution AHR, and (**d**) volume cumulative distribution AHR).

The particle size distribution of the drilling fluid before and after aging was measured, and the results are shown in Figure 8 and Table 2. Before drilling fluid aging, it can be seen from Figure 8a that the particle size distribution diagram shifted to the left, that is, toward the direction of particle size reduction as the LDMS addition increases. The D_10_ (particle size value at 10% of the cumulative volume), D_50_ (median particle size), and D_90_ particle size value at 90% of the cumulative volume) of the bentonite particle size also decreased gradually (Figure 8b and Table 2). After drilling fluid aging, it can be seen from Figure 8c that the particle size distribution diagram also shifted to the left, that is, toward the direction of particle size reduction with the increase in LDMS addition. The peak at 100 μm gradually decreased. When the amount of LDMS was increased to 2 wt%, there was only one peak at about 10 μm in the curve. The overall curve showed a normal distribution, and the particle size distribution was relatively concentrated. Although the D_50_ of the drilling fluid containing 2 wt% LDMS increased suddenly, its D_90_ was only 17.7 μm, indicating that the overall particle size of bentonite particles in the drilling fluid was reduced significantly. At the same time, it can be seen from Table 2 that the particle size of the bentonite particles became smaller after the drilling fluid was aged. Due to the effect of high temperature dehydration, the hydration film on the surface of bentonite particles became thinner under the influence of high temperature, and the interaction force between bentonite particles was reduced. As a result, the bentonite particles changed from an end-to-face card structure to a face-to-face and end-to-end contact structure, and some of the bentonite particles tended to agglomerate together, which was manifested by a peak at 100 μm in the particle size distribution diagram. In the case of LDMS added in the drilling fluid, with the increase in LDMS addition, more and more small molecules of LDMS were adsorbed on the surface of bentonite particles, which maintained the hydration degree of bentonite particles, increased the repulsion between bentonite particles, and increased the content of fine particles in the drilling fluid. Relatively speaking, the smaller bentonite particles have a larger specific surface area and a thicker surface hydration layer, which leads to a higher absolute value of the Zeta potential [33,34].

#### 2.3.2. Effect on the Interlayer Spacing and Interlayer Water Content of Bentonite

**Figure 9:**
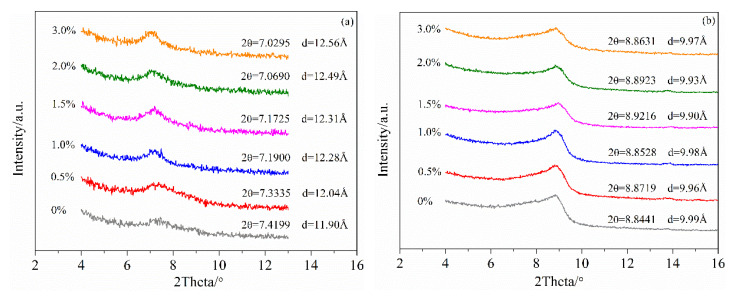
X-ray diffraction pattern of dry bentonite samples treated by a filtrate reducer: (**a**) LDMS; (**b**) Driscal.

The XRD patterns and interlayer spacing (d_001_) results of the dry bentonite treated with different concentrations of filtrate reducers are shown in Figure 9. With the increase in LDMS content, the interlayer spacing of bentonite gradually increased (Figure 9a). In the dry state, the interlayer spacing of original bentonite was 11.90 Å. After the addition of 3 wt% LDMS, the interlayer spacing of LDMS-modified bentonite was enlarged to 12.56 Å, indicating that the small molecular chains of LDMS intercalated into the interlayer of sodium bentonite. However, with the increase in Driscal addition, the interlayer spacing of Driscal-modified bentonite did not change significantly (Figure 9b), indicating that the macromolecular chain of Driscal could not intercalate into the interlayer of bentonite. The LDMS molecular chain has a large number of hydrophilic groups, which can adsorb a large number of water molecules. When the hydrated LDMS molecules intercalated into the interlayer of sodium bentonite, the interlayer adsorbed water content of bentonite increased. The increase in the interlayer water content of bentonite has a good promotion and maintenance effect on the hydration of sodium bentonite [35,36,37].

**Figure 10:**
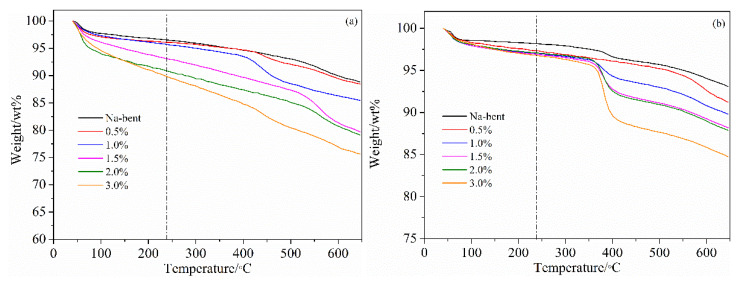
Thermogravimetric curve of bentonite modified by the filtrate reducer: (**a**) LDMS, (**b**), and Driscal.

In order to further understand the adsorption and hydration behavior of small-molecular-weight filtrate reducer LDMS on sodium bentonite, the thermal decomposition results of sodium bentonite modified by different amounts of LDMS and Driscal were obtained and are depicted in Figure 10. The thermal decomposition process of original sodium bentonite after being treated by organic treatment agents can be divided into four stages [38,39]: the first stage is the desorption of bentonite adsorbed water, the second stage is the desorption of water molecules on the surface of hydration ions between bentonite layers, the third stage is the thermal decomposition of organic matter on the bentonite, and the fourth stage is the dehydroxylation of clay minerals. The interlayer adsorbed water of bentonite has an important influence on the hydration of bentonite, so we combined the first and second stages of the bentonite thermogravimetric curve for analysis. Studies have shown [40] that the thermal weight loss of the first and second stages of bentonite is mainly in the range of room temperature to 200 °C. From the TG curve of LDMS (Figure 3), it can be determined that the thermal decomposition temperature of LDMS is 238 °C, so the temperature range of the first and second stages was determined to be 40–238 °C for analysis. From the results in Figure 10a, it can be seen that from the initial temperature to 238 °C, the bentonite lost the adsorbed water and the interlayer bound water. With the increase in LDMS concentration, the weight loss also increased gradually. When the concentration of LDMS was 3 wt%, the weight loss of bentonite was 10.2%. It indicated that LDMS increased the interlayer water content of bentonite after intercalating into the bentonite interlayer. It can be seen from Figure 10b that after Driscal was adsorbed with bentonite, the weight loss of the first and second stages of bentonite also increased. When the amount of Driscal was 3 wt%, the weight loss of bentonite was the highest at 3.2%. From Figure 9b, we can conclude that Driscal cannot intercalate into the interlayer of sodium bentonite, but can only be adsorbed on the surface of bentonite. It indicated that the adsorption and hydration of the filtrate reducer on the bentonite surface slightly increased the content of adsorbed water in bentonite. Moreover, it can be seen from the weight loss of the TG curve that the adsorption amount of the low-molecular-weight filtrate reducer LDMS on bentonite was higher than that of the macromolecule filtrate reducer Driscal under the same addition conditions.

#### 2.3.3. Adsorption Capacity

**Figure 11:**
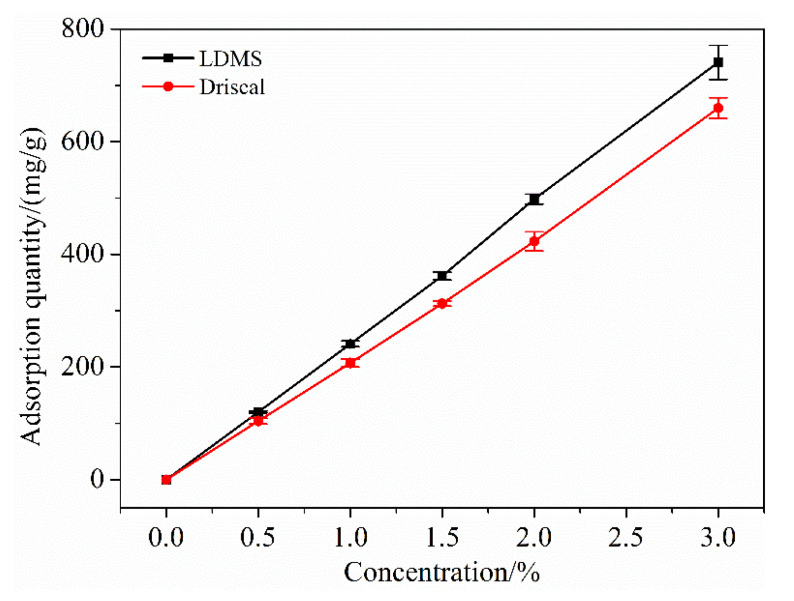
Adsorption quantity of the filtrate reducer on bentonite.

After the drilling fluids were aged at 180 °C, the adsorption amount of the filtrate reducer on bentonite was calculated according to Formula (4), and the results depicted in Figure 11. With the increase in the concentration of the filtrate reducer, its adsorption quantity on bentonite also increased gradually. Additionally, the adsorption quantity of the low-molecular-weight filtrate reducer LDMS on bentonite was higher than that of the macromolecule filtrate reducer Driscal on bentonite. After high temperature aging, LDMS still had a high adsorption quantity on bentonite. When the concentration was 3 wt%, the adsorption quantity of LDMS on bentonite was 740.72 mg/g. The efficient adsorption of LDMS on bentonite maintained a high degree of hydration and good dispersibility of the bentonite particles at high temperature. The filter cake formed by bentonite particles with good dispersibility and small particle size is denser, which can significantly reduce the filtration of the drilling fluid.

#### 2.3.4. Filter Cake Analysis

**Figure 12:**
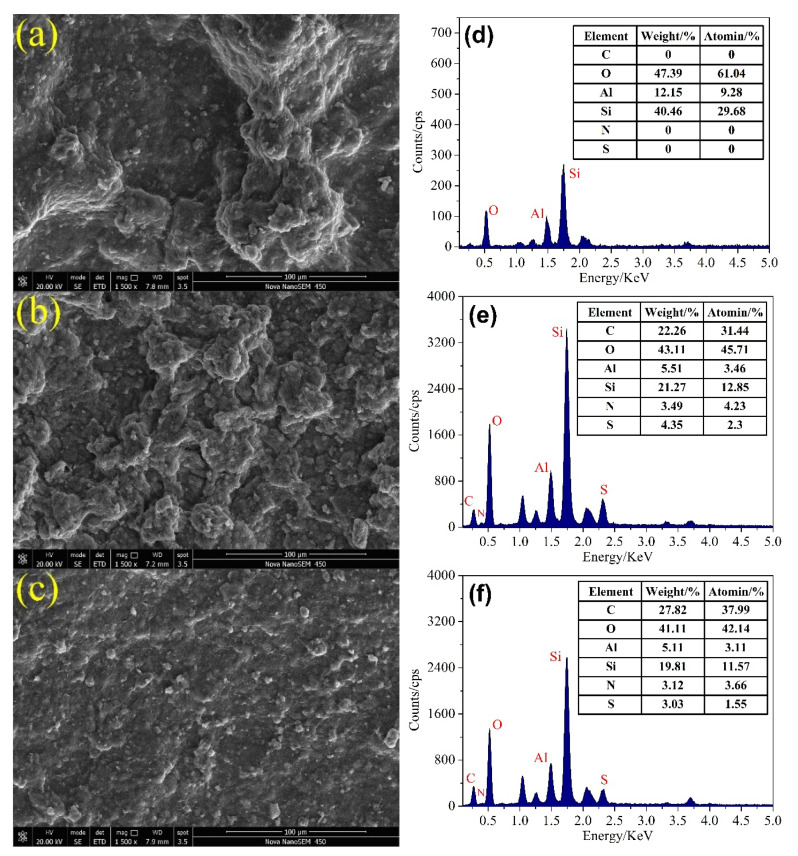
SEM images of the filter cake (1500×) and element content ((**a**,**d**), base fluid; (**b**,**e**), base fluid with 2.0 wt% Driscal; (**c**,**f**), base fluid with 2.0 wt% LDMS).

The drilling fluids were prepared by adding 2.0 wt% LDMS and Driscal to the freshwater base fluid. After aging at 180 °C, the filtrate volume of the drilling fluid was measured according to API standards, and the filter cake was obtained. The microscopic morphology of the dried filter cake was observed by SEM, and the distribution of elements on the surface of the filter cake was measured by EDS. The SEM image and element content determination results of the filter cake of three drilling fluid samples (base fluid, base fluid added with 2.0 wt% Driscal, and base fluid added with 2.0 wt% LDMS) are depicted in Figure 12. In the drilling process, the quality of the filter cake directly affects the filtration performance of the drilling fluid system and drilling safety. Therefore, the quality of the filter cake is also an important evaluation index for the performance of the drilling fluid system [41,42]. The surface of the base fluid filter cake (Figure 12a) was very rough. Additionally, there were obvious large particles and many cracks and holes on the surface of the filter cake. This is due to the high temperature dehydration effect of high temperature on the bentonite particles, resulting in the thinning of the hydration film on the surface of the bentonite particles and the decrease in the repulsion between the particles. Part of the bentonite particles agglomerated and piled up to form large particles. The particle size measurement results showed that the D_10_ of the bentonite particles in the base fluid was 2.19 μm, while the D_90_ was as high as 74.9 μm (Table 2). The loose packing of bentonite particles with a large size difference led to a large amount of drilling fluid filtration. The filter cake of the base fluid with Driscal (Figure 12b) had an uneven surface, and there were large bentonite particles, some of which coalesced with each other. Additionally, there were many microcracks and small pores. There were no obvious cracks and holes on the filter cake of the base fluid with LDMS (Figure 12c). Many fine bentonite particles were closely packed together, and the surface was relatively smooth and flat. After being dissolved in the base fluid, LDMS was firmly adsorbed on the bentonite particles, which maintained the hydration of the bentonite particles and made the bentonite particles have good dispersibility. The overall fine and homogeneous bentonite particles can form a dense and compact filter cake, thereby significantly reducing the filtration of the drilling fluid.

The premise of the effect of the filtrate reducer is that it can be firmly adsorbed on the bentonite particles in a large amount. In order to intuitively feel the adsorption of the filtrate reducer on the surface of bentonite particles, the changes of elements and element contents on the surface of the filter cake were measured by EDS. Since montmorillonite [27,43] is composed of two silicon–oxygen tetrahedra and one aluminum–oxygen octahedron, it can be seen that the surface of the base fluid filter cake contained a large amount of aluminum, silicon, and oxygen elements in Figure 12d. No filtrate reducer was added to the base fluid, so the surface of the filter cake was virtually free of carbon, sulfur, and nitrogen elements. When the filtrate reducer was added to the base fluid, in addition to aluminum, silicon, and oxygen elements, the presence of carbon, nitrogen, and sulfur elements was also observed in Figure 12e,f. This indicated that the filtrate reducer was adsorbed on the bentonite surface. Additionally, the content of carbon element in the filter cake of the drilling fluid added with LDMS was higher than that in the filter cake of the drilling fluid added with Driscal, indicating that the adsorption quantity of the low-molecular-weight filtrate reducer LDMS on bentonite was higher than that of the macromolecular filtrate reducer Driscal on bentonite. This is also an important reason why LDMS is more effective in reducing filtration than Driscal.

#### 2.3.5. Mechanism of LDMS

**Figure 13:**
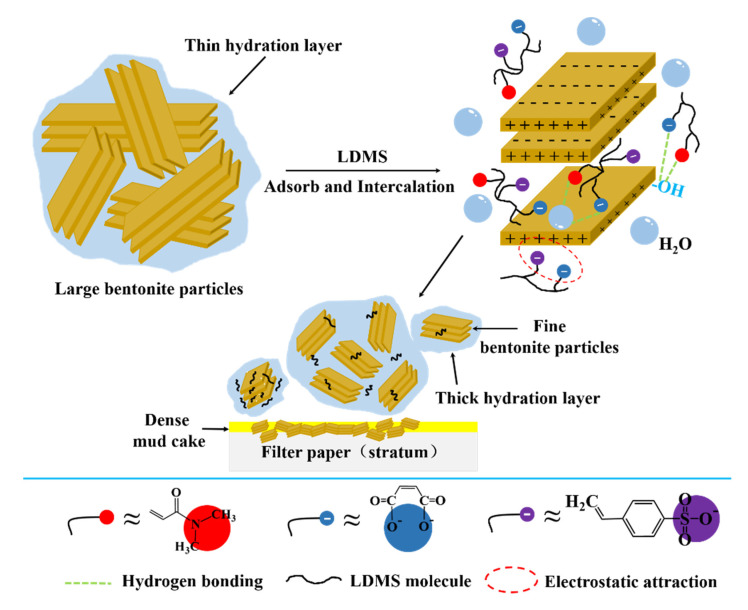
Mechanism of the low-molecular-weight filtrate reducer (LDMS).

Based on the comprehensive analysis of the above experimental results, the mechanism of the low-molecular-weight filtrate reducer LDMS was obtained. In order to better describe the mechanism of LDMS, Figure 13 was drawn for illustration. After the low-molecular-weight filtrate reducer LDMS is added to the drilling fluid, LDMS can be firmly adsorbed on the surface of bentonite particles because its molecular chain contains amide groups as strong adsorption groups. In addition, the LDMS molecular chain contains a large number of negatively charged groups, such as –SO_3_^−^ and –COO^−^, which have good hydration properties. LDMS adsorbs on the surface of bentonite particles under the action of hydrogen bonds and electrostatics, and binds more water molecules on the surface of bentonite particles. On the one hand, the negative charge of the bentonite particles is enhanced so that the repulsion between the bentonite particles increases, which is beneficial for the bentonite particles to maintain a small particle size. On the other hand, the thickness of the bentonite hydration layer is increased, and the colloidal stability of the drilling fluid is improved. At the same time, the small molecular chain of LDMS contains rigid groups, and compared with high-molecular-weight long-chain polymers, the small molecular chain is not easy to break, so it has better high temperature stability. Additionally, small molecular chains can intercalate into the interlayer of sodium bentonite. Under the action of hydrated ions of the LDMS molecular chain, the interlayer water content and hydration degree of bentonite are increased, and the resistance to high temperature dehydration is enhanced. In general, the unique chemical structure of LDMS, including rigid small molecular chain, strong adsorption groups, and highly negatively charged hydration groups, makes it have excellent temperature resistance and filtration reduction performance.

## 3. Conclusions

In this work, a novel high-temperature-resistant filtrate reducer (i.e., LDMS) with low molecular weight was synthesized using DMAA, MAH, and SSS as reaction monomers. TGA experiment showed that the polymer LDMS has good thermal stability, the initial decomposition temperature is as high as 238 °C, and it has a good application prospect in a high-temperature-resistant drilling fluid gel system. At the same time, unlike the commonly used macromolecular polymer filtrate reducers, LDMS does not significantly increase the viscosity and yield point of a drilling fluid gel system due to its low molecular weight (M_w_ is 1325 Dalton). As a result, when 2 wt% LDMS was added to the base fluid, the apparent viscosity of the drilling fluid gel system was 8.5 mPa·s and the API filtration loss was only 8.0 mL after high temperature aging at 210 °C. In addition, the mechanism of LDMS was further studied. It was confirmed that due to the unique chemical structure of LDMS, including a rigid small molecular chain, strong adsorption groups and highly negatively charged hydration groups enabled it to be adsorbed on the surface of bentonite particles in large quantities and intercalated into the interlayer of bentonite. Thus, the hydration degree of the bentonite particles and the colloidal stability of the drilling fluid gel system were improved, the aggregation of the bentonite particles was inhibited, the content of fine particles in the drilling fluid gel system was maintained, and a compact filter cake was formed, which significantly reduced the filtration of the drilling fluid. Overall, this work will facilitate the application of a low-molecular-weight polymer filtrate reducer in high-temperature-resistant water-based drilling fluid gel systems.

## 4. Materials and Methods

### 4.1. Materials

N, N-dimethylacrylamide (DMAA, GC), maleic anhydride (MAH, AR), and sodium p-styrene sulfonate (SSS, AR) were purchased from Aladdin Reagent Co., Ltd. (Shanghai, China). Ammonium persulfate (AR), anhydrous ethanol, and sodium hydroxide (AR) were purchased from Sinopharm Chemical Readditive Co., Ltd. (Shanghai, China). Driscal D Polymer (Driscal) was purchased from Chevron Phillips Chemical Company (Wikiwand, TX, USA). The sodium bentonite used for water-based drilling fluids (WBDFs) was purchased from Huaian Tengfei Bentonite Development Co., Ltd., China (Huaian).

### 4.2. Synthesis and Characterization of LDMS

#### 4.2.1. LDMS Synthesis

**Scheme 1:**
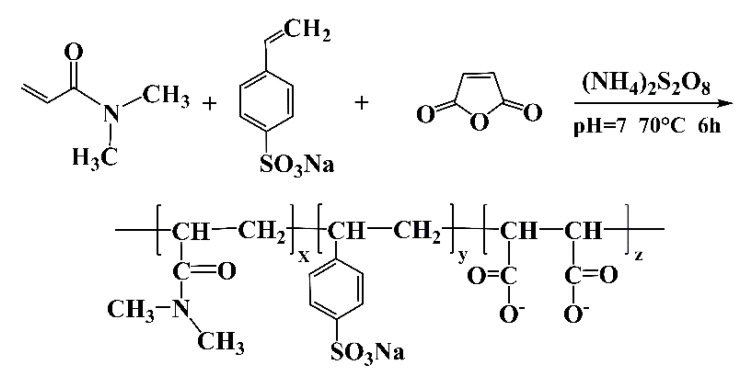
The synthesis procedure and molecular structural of LDMS.

In total, 9.9 g DMAA, 9.8 g MAH, and 10.3 g SSS were mixed evenly in a beaker containing 75 g deionized water. The beaker was heated in a water bath at 40 °C. The reaction monomer was completely dissolved by constant stirring, and the pH of the solution was adjusted to 7 with a dilute NaOH solution. Next, the solution was transferred from the beaker to the flask. When the water bath was heated to 70 °C, 0.15 g ammonium persulfate was added to the flask. The reactants were reacted under nitrogen atmosphere for 6 h to obtain a viscous liquid product. The product was purified with acetone and ethanol, dried under vacuum at 50 °C, and pulverized to obtain a low-molecular-weight filtrate reducer, namely, LDMS. The synthesis process and possible molecular structure of LDMS are shown in Scheme 1.

#### 4.2.2. LDMS Characterization

(1) Fourier-transform infrared (FT-IR)

The synthetic product was characterized by the potassium bromide (KBr) tabletting method using an IRTRacer-100 infrared spectrometer (Shimadzu Corporation, Kyoto, Japan). A small amount of sample was mixed well with KBr in a mortar and ground thoroughly, and the ground powder was pressed into a complete and transparent sheet using a tablet press. The scanning wavelength range was from 500 to 4000 cm^−1^, the resolution ratio was 4 cm^−1^, and the sample scanning times were 32.

(2) Nuclear magnetic resonance hydrogen spectrum (^1^H-NMR)

To further characterize the molecular structure of LDMS, its NMR hydrogen spectrum was measured using an Avance NEO 400 MHz NMR spectrometer (Bruker, Berlin, Germany). The signal was collected by dissolving 3 mg of sample in heavy water and cycling 16 times.

(3) Thermogravimetric analysis (TGA)

The thermal stability of LDMS was measured using a thermal analyzer (TGA550, Mettler Toledo Co. Shanghai, China) in a N_2_ environment. The experimental temperature range was 50.0 to 800.0 °C, and the heating rate was 10.0 °C/min.

(4) Gel permeation chromatography (GPC)

The relative molecular mass distribution of the filtrate reducer samples LDMS and Driscal was determined by gel chromatography using a Waters 1515 GPC type gel chromatograph (Waters Corporation, Milford, MA, USA). The mobile phase of the test was 0.1 mol/L aqueous sodium nitrate solution, and the standard was polyethylene glycol (PEG).

### 4.3. Drilling Fluid Preparation and Performance Test

Sixteen grams of bentonite, 0.56 g of sodium carbonate, and 400 mL of distilled water were added into a high-speed mixing cup and stirred at 10,000 rpm for 20 min, and then maintained for hydration at room temperature for 24 h to derive the base fluid. The filtrate reducer sample was added to the base fluid and stirred at 4000 rpm for 20 min to obtain the drilling fluid sample to be tested.

The rheology of the drilling fluids was tested by a six-speed rotary viscosimeter (ZNN-D6, Haitongda Co., Ltd., Qingdao, China). The following equations calculate the apparent viscosity (AV), plastic viscosity (PV), and yield point (YP):

(1) $$\mathrm{AV}=\frac{\theta_{600}}{2}\;(\mathrm{mPa}\cdot s)$$

(2) $${\mathrm{PV}=\theta }_{600}-{\;\theta }_{300}\;(\mathrm{mPa}\cdot s)$$

(3) $${\mathrm{YP}=(\theta }_{300}-\;\mathrm{PV})/2\;(\mathrm{Pa})$$

where θ_600_ and θ_300_ are the values of the rotary viscometer at the corresponding speed.

According to American Petroleum Institute (API) standards, the API filtration of drilling fluids was measured by a medium pressure filtration meter (SD, Qingdao Tongchun Petroleum Instrument Co., Ltd. Qingdao, China) at a fixed pressure of 100 psi for 30 min. The drilling fluids were aged in the aging kettle for 16 h. The rheological parameters and API filtration were measured before and after the aging experiments, using a high-temperature and high-pressure filtration apparatus (GGS42-2, Qingdao Tongchun Petroleum Instrument Co., Ltd.), maintaining a fixed pressure difference of 3.5 Mpa, and measuring the high-temperature and high-pressure (HTHP) filtration of the drilling fluids for 30 min at the corresponding aging temperatures.

### 4.4. Zeta Potential and Particle Size Analysis

The Zeta potential value of the drilling fluid samples was measured by a nanoparticle potentiometer (Zetasizer Nano Z, Malvern Instruments Co., Ltd., Malvern, UK). The particle size distribution of the drilling fluids was determined using a laser particle size analyzer (Mastersizer 3000, Malvern Instruments Co., Ltd.).

### 4.5. Effect of Filtrate Reducer on Bentonite

The drilling fluid samples were prepared by adding different contents of the filtrate reducer to the base fluid. The drilling fluids were cooled and centrifuged after aging at 180 °C for 16 h. The bentonite obtained by centrifugation was rinsed three times with deionized water, dried at 105 °C for 4 h, and ground through a 200 mesh sieve to obtain the bentonite treated with filtrate reducers. The lattice spacing of bentonite was measured by X-ray diffraction (XRD) using an X-ray diffractometer (XPert Powder, PANalytical B.V. Almelo, Netherlands). The experimental test used a Cu target, the test voltage was 40 kV, and the test current was 40 mA. Thermogravimetric analysis of the bentonite was performed using a thermogravimetric analyzer (TGA550, Mettler Toledo Co.) with a nitrogen atmosphere. The heating rate was 10.0 °C/min.

### 4.6. Adsorption Capacity of Filtrate Reducer on Bentonite Surface

The filtrate was collected when measuring the HTHP filtration volume of drilling fluids containing different contents of the filtrate reducer at 180 °C. The total organic carbon content (TOC) in the filtrate was measured by a total organic carbon tester (multi N/C 3100, Analytik Jena AG, Jena, Germany). The adsorption amount of the filtrate reducer on the drilling fluid bentonite was calculated by the following equation [44]:

(4) $$K=\frac{m_{1}-{\;m}_{2}\times P/L}{m_{0}}$$

where K is the adsorption amount of the filtrate reducer on bentonite (mg/g); m_0_ is the mass of bentonite in the drilling fluid (g); m_1_ is the mass of the filtrate reducer in the drilling fluid (g); m_2_ is the mass of the filtrate (g); L is the mass percentage of elemental carbon in the molecule of the filtrate reducer (%); and P is the amount of carbon element in the filtrate (mg/L), which is the measured value of the TOC experiment.

### 4.7. Microscopic Morphological Analysis of Filter Cake

The filtrate volume of the drilling fluid was measured according to API standards, and the filter cake was obtained after the measurement. After the filter cake was dried, its microscopic morphology was observed with scanning electron microscopy (SEM, Nova NanoSEM 450, FEI Company, Hillsboro, OR, USA), and the distribution of elements on the surface of the filter cake was studied by an energy dispersive spectrometer (EDS, Inca X-Max 50, Oxford Instrument Technology Co., Ltd., Concord, MA, USA).

## Figures and Tables

**Table 1:** Molecular weight and polydispersity of LDMS and Driscal.

Sample	Mn	Mw	Mz	Polydispersity
LDMS	1145	1325	1488	1.157
Driscal D Polymer	108,114	626,763	1,786,768	5.797

**Table 2:** Particle size of the drilling fluid before and after aging.

Concentration/%	D_10_/μm		D_50_/μm		D_90_/μm	
	BHR	AHR	BHR	AHR	BHR	AHR
0 (Base fluid)	2.42	2.19	20.0	9.35	113	74.9
0.5	2.41	2.18	19.0	8.94	106	69.3
1	2.41	1.99	18.5	6.73	105	30.2
1.5	2.35	1.98	16.7	6.61	102	33.9
2	2.33	2.23	16.6	7.38	99.6	17.7
3	2.24	1.74	13.4	4.86	88.0	14.4

## Data Availability

Not applicable.
